# Surgeon-General Jeffery Allen Marston

**Published:** 1911-04-08

**Authors:** 


Surgeon-General Jeffery Allen Marston, Honorary Surgeon to the King, who died last week in London, at the age of seventy-nine, obtained his M.D. degree at the University of St. Andrews in 1854. He took his M.R.C.P. in 1887, and became F.R.C.S. Eng. in 1888. He entered the Army in 1854, and was present at the battle of Tel-el-Kebir. He was mentioned in despatches and received several decorations. General Marston was formerly head of the Army Sanitary Branch of the Medical Department of the War Office, a member of the Army Sanitary Committee, President of the Army Medical Board, and a member of the Indian Medical Board. He was the author of several Army Medical Reports on sanitary work and on various fevers. He was created a C.B. in 1887, and retired from the Army in 1889.

